# Tumor Microenvironment in Melanoma—Characteristic and Clinical Implications

**DOI:** 10.3390/ijms26146778

**Published:** 2025-07-15

**Authors:** Hubert Sikorski, Michał Aleksander Żmijewski, Anna Piotrowska

**Affiliations:** Department of Histology, Faculty of Medicine, Medical University of Gdańsk, Dębinki 1 Str., 80-211 Gdańsk, Polandmichal.zmijewski@gumed.edu.pl (M.A.Ż.)

**Keywords:** melanoma, tumor microenvironment, cancer/melanoma-associated fibroblasts (CAFs), macrophages, tumor-infiltrating lymphocytes (TILs), dendritic cells, metastases, immune checkpoint inhibitors (ICI)

## Abstract

Cutaneous melanoma is an aggressive cancer with an increasing incidence worldwide, highlighting the need for research into its pathogenesis. The tumor microenvironment (TME) plays a critical role in melanoma progression and consists of cellular components and an extracellular matrix (ECM) rich in cytokines and signaling molecules. The most abundant stromal cells within the TME are cancer-associated fibroblasts (CAFs), which remodel the ECM and modulate immune responses. Among immune cells, tumor-associated macrophages (TAMs) predominate, and their polarization toward the M2 phenotype supports tumor progression. Tumor-infiltrating lymphocytes (TILs) have diverse functions, including cytotoxic T-cells, helper T-cells that modulate immune response, B-cells forming tertiary lymphoid structures (TLS), and regulatory T-cells with immunosuppressive properties. Dendritic cells (DCs) also play a complex role in the TME. A notable subpopulation are mature regulatory dendritic cells (mregDCs), which contribute to immune evasion. All of these TME components may drive tumorigenesis. Advancements in melanoma treatment—including immunotherapy and targeted therapies—have significantly improved outcomes in advanced-stage disease. In parallel, emerging approaches targeting the tumor microenvironment and gut microbiome, as well as personalized strategies such as neoantigen vaccines and cell-based therapies, are under active investigation and may further enhance therapeutic efficacy in the near future.

## 1. Introduction

According to the World Health Organization, currently even one in every three cancers diagnosed is a skin cancer [1]. Skin neoplasms are the most common malignant tumors in Caucasians, and the lifetime risk of developing the disease in this population exceeds 20% [2]. Basal cell carcinoma (BCC) dominates among skin cancers, accounting for about 80% of cases. The second most common type is squamous cell carcinoma (SCC), representing approximately 15–20% of cases [2]. Other forms of skin carcinomas, such as Merkel-cell carcinoma, known also as neuroendocrine carcinoma of skin, or sebaceous carcinoma, are much less common [2].

**Figure 1:**
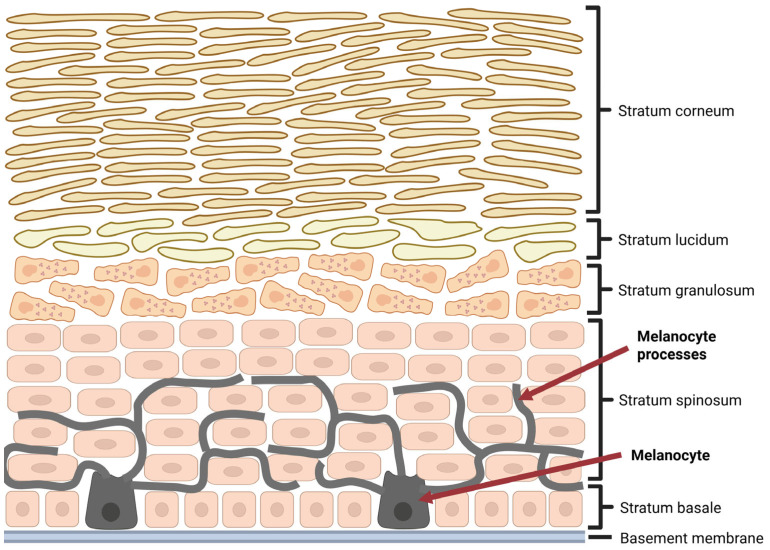
Localization of melanocytes in epidermis. Created in BioRender. Sikorski, H. (2025) https://app.biorender.com/illustrations/680f42c88ad34f409d0785d3 (accessed on 11 July 2025).

Cutaneous malignant melanomas constitute only a minor percentage of all skin neoplasms, as they represent roughly about 1–4% of cases [3,4,5]; however, they are responsible for the vast majority of deaths caused by cancers of this location [6,7,8,9]. Moreover, since 1975, melanoma incidence has increased significantly compared to other neoplasms [10,11]. Although this alarming trend recently appears to be stabilized, at least in men, melanoma ranks fifth among the most frequently diagnosed types of cancer in both sexes, excluding basal cell and squamous cell skin cancers, as prognosed by the American Cancer Society [11]. Epidemiological data indicate that, despite a higher absolute number of melanoma cases in men, the proportional incidence relative to all cancer diagnoses is somewhat higher in women. This difference is primarily attributable to the distinct cancer epidemiology patterns between sexes [11]. Interestingly, melanoma incidence exhibits clear age- and sex-related variations, with women more commonly affected at a younger ages, whereas incidence increases in men after the age of 50, reflecting differential exposure to risk factors as well as behavioral and biological differences [11]. Although sex differences in melanoma survival have been described in the literature, their causes remain unclear [12,13,14]. An analysis of 1753 cases from the GEM study showed that the female survival advantage is mainly due to an indirect effect of sex mediated by clinicopathologic tumor features—such as age at diagnosis, Breslow thickness, ulceration, mitotic rate, and tumor site—rather than a direct biological effect of sex [13]. However, other studies have reported female sex as an independent prognostic factor in melanoma survival, suggesting that biological sex itself may directly influence patient outcomes [14]. Malignant melanoma is a highly aggressive skin neoplasm, originating from neuroectodermal melanin-producing cells, known as melanocytes, found predominantly among the cells of the basal layer of epidermis (Figure 1) [4,15,16].

It should be noted, however, that melanocytes are also found in the eye, inner ear, and leptomeninges [3,17]. Ultraviolet (UV) radiation, both natural and artificial, is considered the most important environmental risk factor for the development of melanoma [10,18,19]. Other melanoma risk factors include permanent mechanical or chemical irritation, low pigment content in the skin, as well as genetic predispositions, such as familial atypical mole syndrome (FAMS) [10]. Considering the above, protection against excessive ultraviolet radiation is one of the most important elements of melanoma prevention [10]. Melanomas developing at sites permanently exposed to UV radiation, such as head and neck, most often appear in older patients, in the seventh decade of life, whose cumulative exposure to UV radiation has reached a high level over the years [20]. Interestingly, melanomas developing in body regions only intermittently exposed to long-term UV radiation, such as the trunk, are usually detected in younger patients, between the third and sixth decades of life, and are associated with a history of cutaneous sunburns [20]. It is believed that sunburns incurred in childhood and adolescence increase the risk of cutaneous melanoma, especially in fair-skinned people with blond or red hair with multiple nevi [18].

The vast majority of melanomas are sporadic, while in approximately 5–12% of melanoma patients, a family history of the disease is observed [9,18]. It should be emphasized that melanomas have the highest mutation frequency of all cancers analyzed [21]. In the familial form of melanoma, mutations in the CDKN2A gene are commonly found, which encodes two separate proteins, p14ARF and p16INK4a, important tumor suppressors that regulate the G1 cell cycle checkpoint and stabilize the expression of p53 [9,18]. In sporadic melanomas, the majority of mutations are acquired in the genes encoding proteins of the MAPK (mitogen-activated protein kinase) pathway. It is estimated that these mutations are detected in 70% of melanomas [9]. The mitogen-activated protein kinase pathway is responsible for integration of signals received by cells from the external environment in the form of growth factors with the regulation of intracellular processes such as growth, cellular division, differentiation, and apoptosis [22]. The consequence of mutations in the genes encoding the MAPK pathway components is its permanent activation, leading to uncontrolled division of cancer cells [9,17,21]. For example, roughly 50% of melanomas are characterized by activating BRAF mutations, the most common of which is the V600E mutation, accounting for 70–88% of all BRAF mutations [9,21]. The substitution of the hydrophobic amino acid, valine, with hydrophilic amino acid, glutamic acid, causes constitutive activation of the catalytic domain of BRAF serine/threonine protein kinase, resulting in a 500-fold increase in kinase activity compared with wild-type BRAF kinase [23]. Interestingly, mutations in the gene encoding the BRAF protein are predominantly observed in melanomas arising in body regions only intermittently, rather than persistently, sun exposed, with a superficial spreading melanoma phenotype [9,21]. It is further estimated, that another 15–20% of melanomas have NRAS mutations, while 2% have CKIT mutations, the latter most commonly found in mucosal melanomas [9,21]. In over 80% of cases, NRAS displays a decreased GTPase activity as a result of a point mutation, implying that both MAPK and PI3K (phosphoinositide 3-kinases) signaling pathways remain activated [23].

Although for a long time researchers primarily focused on cancer cells, the role of stromal cells is no longer seen merely as a passive presence in the tumor microenvironment (TME); on the contrary, they are increasingly recognized as key players influencing disease progression and the development of oncological therapies [5,24]. This review aims to comprehensively summarize the current understanding of the tumor microenvironment in melanoma, highlighting its key characteristics and exploring the clinical implications, including potential therapeutic strategies targeting stromal and immune components.

## 2. Melanoma Microenvironment—Characteristic and Clinical Implications

**Figure 2:**
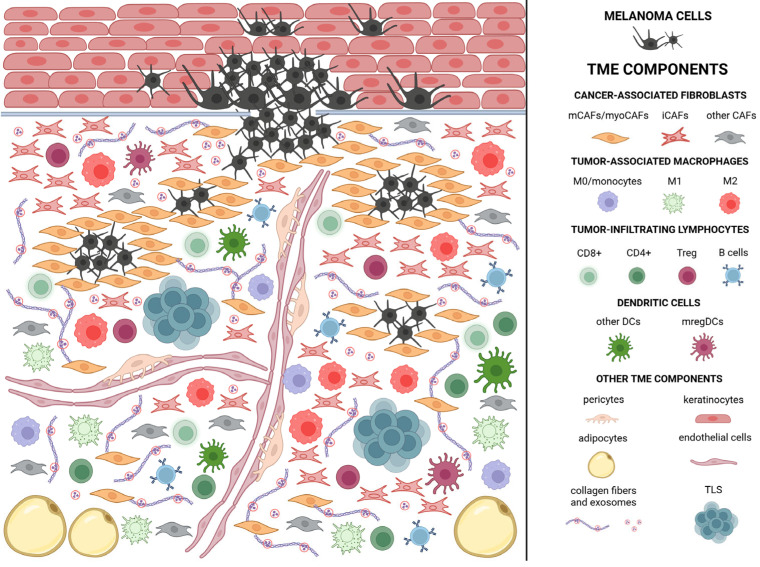
Melanoma microenvironment. Created in BioRender. Sikorski, H. (2025) https://app.biorender.com/illustrations/681ba19b77ac8eff499a4020 (accessed on 11 July 2025). The figure schematically represents the composition of the tumor microenvironment in melanoma. Numerous CAFs (cancer-associated fibroblasts) are visible, constituting the most abundant cellular component of the tumor microenvironment. Matrix CAFs (mCAFs) surround tumor cells, forming a barrier to immune cell infiltration. They also contribute to the production of the extracellular matrix and the tensioning of its structure. Immunomodulatory CAFs (iCAFs), in turn, secrete factors that promote the acquisition of a tolerogenic/immunosuppressive phenotype by immune cells. For this reason, they were presented in close proximity, forming clusters to illustrate their mutual interactions. Immune cells, as well as iCAFs that exhibit pro-tumorigenic properties, are marked in shades of red. Particular attention should also be paid to the dispersed exosomes produced by tumor cells, which play a key role in the recruitment of tolerogenic cells. These exosomes bind to collagen, ensuring their persistent presence in the tumor microenvironment. The occurrence of TLS (tertiary lymphoid structures) structures is also symbolically indicated, although their cellular composition is more complex and described in detail later in the article.

Advances in oncology research and an increasingly precise understanding of the mechanisms and interactions within tumors have drawn attention to the critical role of the tumor microenvironment at all stages of tumorigenesis. The tumor microenvironment is hypoxic, acidic, and rich in cells, in addition to cancer cells. It comprises stromal cells, with cancer-associated fibroblasts (CAFs) being particularly significant; various types of immune cells; and non-cellular components such as blood and lymphatic vessels, as well as the extracellular matrix with embedded cytokines and other signaling molecules (Figure 2) [25]. The proportions of cells comprising the tumor microenvironment and their interactions may vary depending on the tumor type, as well as across different stages of development of the same tumor [26]. Melanoma cells produce exosomes that can be stored in the tumor microenvironment through interactions with collagen and likely other extracellular matrix (ECM) components [27]. Furthermore, in vitro studies showed a gradual decrease in the amount of soluble collagen during the progression of cell cultures, indicating active extracellular matrix remodeling and partial collagen degradation is essential for tumor progression [28]. A persistent presence of specific molecules within the tumor microenvironment stimulate various cell types to adopt a pro-tumorigenic phenotype [27]. Thus, a substantial portion of interactions within the tumor microenvironment (TME) may occur without direct cell-to-cell contact [29].

### 2.1. Cancer-Associated Fibroblasts/Melanoma-Associated Fibroblasts (CAFs)

Fibroblasts are cells responsible for production and modification of the extracellular components of the connective tissue. In a healthy skin, two populations of fibroblasts can be distinguished: reticular and papillary fibroblasts. Both populations become activated and participate in the wound healing process; however, reticular fibroblasts migrate to the wound area during the early stages of healing, whereas papillary fibroblasts play a key role in the final stage of this process [30]. Fibroblasts are responsible for the production of collagen and fibronectin (Fn) during tissue repair. Transforming growth factor beta (TGF-β) signals derived from macrophages play a crucial role in ECM remodeling by promoting the differentiation of fibroblasts into myofibroblasts. This transition results in enhanced collagen deposition and increased mechanical tension within the extracellular matrix [31].

During tumor formation, fibroblasts located near cancer cells become activated and transform into cancer-associated fibroblasts (CAFs), with reticular fibroblasts showing a greater tendency to undergo this process compared to papillary fibroblasts in the case of skin cancers [25,30]. Aged male fibroblasts exhibit chronic oxidative stress, impaired DNA repair capacity, and secrete factors that enhance melanoma cell invasiveness [32]. These features may facilitate their conversion into CAFs [33]. In contrast, female fibroblasts more effectively neutralize reactive oxygen species and maintain better DNA repair capacity [32]. CAFs are the most abundant cell groups within the tumor microenvironment (TME). The transition from normal fibroblasts to CAFs primarily occurs through epigenetic changes and the activation of specific transcription factors, while significant genomic alterations are not observed [25]. In melanoma, the activation of the CAFs phenotype in normal fibroblasts is driven by melanoma cells secreting: interleukin-1 beta (IL-1β), interleukin-6 (IL-6), interleukin-8 (IL-8), transforming growth factor beta (TGF-β), platelet-derived growth factor (PDGF), fibroblast growth factor 2 (FGF-2), fibroblast growth factor 19 antisense (FGF-19as), microRNA-211 (miR-211), and microRNA-155 (miR-155). The presence of exosomes derived from cancer cells also plays a crucial role [34].

CAFs can arise through several potential pathways: from resident fibroblasts, from bone marrow-derived mesenchymal stem cells (BM-MSCs), from epithelial and endothelial cells via epithelial-to-mesenchymal transition (EMT), and endothelial-to-mesenchymal transition (EndMT), respectively; from macrophages through macrophage–myofibroblast transition (MMT); as well as from adipocytes or pericytes. The epithelial origin is particularly significant in the context of skin cancers, as increased expression of EMT markers in these cancer types correlates with increased tumor malignancy [25].

CAFs can be identified using intracellular biomarkers such as alpha-smooth muscle actin (αSMA); fibroblast-specific protein 1 (FSP-1); vimentin; surface markers including fibroblast activation protein (FAP), podoplanin, platelet-derived growth factor receptor alpha, and beta (PDGFRα and PDGFRβ); extracellular markers like lumican, decorin, and collagen type I alpha 1/2 (COL1A1/2)). Not all of these markers are present on activated CAFs, and various combinations, along with other markers, may indicate the origin and functional role of a given population of the cells [35]. For example, increased expression of the podoplanin gene can lead to changes in the actin cytoskeleton, resulting in enhanced cell mobility and reduced adhesive capabilities [27]. In the case of melanoma, the following activation markers should also be noted: osteonectin, desmin, periostin, cluster of differentiation 90/thymus cell antigen 1 (CD90/THY1), and neuron-glial antigen-2 (NG2) [34]. A very recent publication demonstrated that the combination of two markers—actin alpha 2 (ACTA2) and fibroblast-activation protein (FAP)—is sufficient to isolate all CAFs from the tumor cell population. However, the drawback of this method is that vascular smooth muscle cells (vSMCs) also express those markers [36].

Normal fibroblasts can inhibit tumor development by secreting interleukin-6 (IL-6), interleukin-15 (IL-15), transforming growth factor beta (TGF-β), pigment epithelium-derived factor (PEDF), tumor necrosis factor alpha (TNF-α), interferon gamma (IFNγ), and whey acidic protein four-disulfide core domain 1 (WFDC1) [30]. In contrast, activated CAFs in melanoma secrete a range of factors involved in extracellular matrix (ECM) remodeling—matrix metalloproteinases (MMPs), tissue inhibitor of metalloproteinases 1 (TIMP1), COL1A1/2, a disintegrin and metalloproteinase 9 (ADAM9), and hyaluronan-binding protein 1 (HAPLN1), promoting melanoma cell migration and invasion—insulin-like growth factor 1 (IGF-1), vascular endothelial growth factor A (VEGF-A), FGF-2, stromal cell-derived factor 1 (SDF-1), interleukin-6 and 8 (IL-6/8), chemokine C-C motif ligand 2 (CCL-2), chemokine C-X-C motif ligand 12 (CXCL12), and connective tissue growth factor (CTGF), enhancing cancer cell viability—insulin-like growth factor 1 (IGF-1), hepatocyte growth factor (HGF), VEGF-A, FGF-2, CCL-2, and IL-8, modulating immune responses—TGF-β, CCL2, IL-6, granulocyte-macrophage colony-stimulating factor (GM-CSF), matrix metalloproteinases (MMPs), prostaglandin E2 (PGE2), cyclooxygenase 2 (COX-2), chemokine C-X-C motif ligand 5 (CXCL5), and programmed death ligand 1 and 2 (PDL1/2). Additionally, these factors also contribute to therapy resistance [5,34]. CAFs exhibit elevated expression of FGF2 compared to normal fibroblasts. This growth factor exerts its effects through interaction with fibroblast growth factor receptor 1 (FGFR1), promoting cancer cell migration and invasion, as well as stimulating the expression of Cyclin D1. Both FGF2 neutralization and FGFR1 silencing eliminate these effects [37]. CPL304110 (an FGFR inhibitor) reduces the viability of melanoma cells and, depending on the cell line, induces either G0/G1 cell cycle arrest or increases the proportion of apoptotic cells. There is evidence indicating a complex interaction between FGFR and vitamin D signaling pathways, affecting cell cycle phase distribution, the expression of FGFR1 and FGFR2 receptors, and activation of the extracellular signal-regulated kinases 1/2 (ERK1/2) pathway. This relationship warrants further investigation, as modulation of the vitamin D pathway may significantly influence the biological effects of FGFR inhibitors [38]. It is worth noting that in a study conducted on patient-derived melanoma cultures, melanoma cells exhibited preferential growth on a fibroblast layer. Despite the use of geneticin to eliminate fibroblasts, their population persisted and expanded over subsequent passages. These findings suggest that direct contact with fibroblasts supports melanoma cell survival. The importance of such direct cell-to-cell interactions is further underscored by the observation that culturing melanoma cells in medium containing CAF-derived supernatant was insufficient to sustain their growth [39]. Fibroblasts play a crucial role in the structural organization of tumors. This is demonstrated by an experiment in which in vitro cultures of melanoma cells combined with macrophages formed loose aggregates, whereas cultures additionally containing fibroblasts developed compact, three-dimensional structures resembling those observed in vivo. This effect was partially dependent on the timing of fibroblast introduction, as in the condition where fibroblasts were added at a later stage of culture, the resulting spheroids exhibited less defined and less cohesive boundaries compared to those formed when fibroblasts were present from the onset [28]. An example of TME remodeling is the tensioning of collagen fibers. Such a role of CAFs is suggested by studies utilizing a 3D model, in which regions containing fibroblasts exhibited more aligned collagen fiber organization and increased fiber thickness [28]. CAFs generally exhibit pro-inflammatory activity that enhances the aggressiveness of many cancers, including melanoma. In melanoma, this activity in CAFs is promoted by increased expression of IL-1β, IL-6, IL-8, C-X-C motif chemokine ligand 8 (CXCL8), and various types of metalloproteinases [5,27,36]. It is worth noting that individual factors can have either anti-tumor or pro-tumor effects depending on the involvement of other regulators and the stage of tumor development. This is the case for IL-6 and TGF-β, which initially have inhibitory effects, but at advanced stages, they exert pro-tumorigenic effects [30].

Agnes Forsthuber and colleagues identified four subtypes of CAFs present in skin cancers: matrix CAFs (mCAFs), immunomodulatory CAFs (iCAFs), myofibroblast-like cancer-associated fibroblasts (myoCAFs), and an unclassifiable CAF subtype (ucCAFs). mCAFs are characterized by increased expression of extracellular matrix components such as collagens (COL1A1, COL1A2, COL3A1), lumican (LUM), periostin (POSTN), and tenascin-C (TNC). This subtype is primarily present in less aggressive tumors, such as basal cell carcinoma (BCC) and well-differentiated squamous cell carcinoma (SCC). mCAFs also express transcription factors such as C-X-C motif chemokine ligand 5 (CXCL5), transcription factor 4 (TCF4), twist-related protein 1 (TWIST1), and twist-related protein 2 (TWIST2), as well as anti-inflammatory signaling factors like potassium channel-interacting protein 3 (KCNIP3). mCAFs form a dense network of collagen fibers around the tumor, which acts as a physical barrier, restricting infiltration of immune cells into the tumor core. iCAFs exhibit significantly higher expression levels of genes associated with matrix remodeling, such as matrix metalloproteinase 1 (MMP1) and 3 (MMP3), as well as pro-inflammatory cytokines, including interleukin-6 (IL-6) and C-X-C motif chemokine ligand 8 (CXCL8), and molecular regulators of immunosuppression, such as indoleamine 2,3-dioxygenase 1 (IDO1). The expression of these genes indicates that iCAFs are involved in modulation of the immune response and support tumor invasion. The presence of iCAFs is particularly prominent in melanoma samples and in aggressive tumors, such as late-differentiated SCC and infiltrative BCC. Compared to mCAFs, iCAFs show elevated expression of transcription factors related to the immune response, such as signal transducer and activator of transcription 1 (STAT1), interferon regulatory factor 1 (IRF1), interferon regulatory factor 9 (IRF9), and AT-rich interactive domain-containing protein 5A (ARID5A). myoCAFs represent a subset of activated fibroblasts that shows the expression of actin alpha smooth muscle (ACTA2), collagen type I alpha 1 (COL1A1), and pericyte markers such as regulator of G protein signaling 5 (RGS5), inwardly rectifying potassium channel 8 (KCNJ8), and melanoma cell adhesion molecule (MCAM). RGS5+ myofibroblasts are present in all skin tumor subtypes, which may suggest their general role in fibrotic and neoplastic processes. The shared markers with the vascular smooth muscle cells (vSMC) suggest their important function in perivascular tissues. ucCAFs represent a small population of fibroblasts with an ambiguous gene expression profile [36].

**Figure 3:**
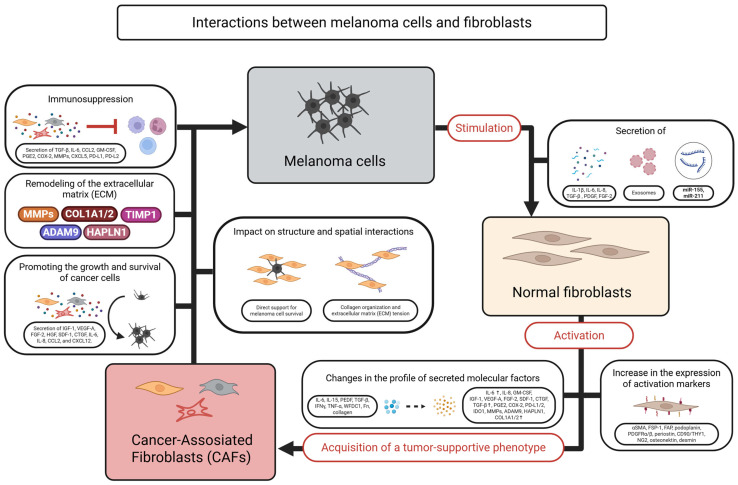
Schematic representation of the interactions between melanoma cells and fibroblasts within the tumor microenvironment. Created in BioRender. Sikorski, H. (2025) https://app.biorender.com/illustrations/6811333a9b48cfcbde3c43a8 (accessed on 11 July 2025).

The scheme presented in Figure 3 illustrates the key interactions between melanoma cells and fibroblasts, which play a crucial role in shaping the tumor microenvironment.

### 2.2. Tumor-Associated Macrophages (TAMs)

The most abundant types of immune cells in the tumor microenvironment (TME) are macrophages. These cells have the ability to perform phagocytosis and present antigens to T-lymphocytes, enabling them to contribute to both innate and adaptive immune responses [40]. Macrophages infiltrate the TME and are followed by T-cells. In the early stages of melanoma development in a mouse model, they serve as the cornerstone of the immune response [28].

Macrophages can develop through two independent pathways: from bone marrow myeloid progenitors or from yolk sac and fetal liver cells during embryogenesis. The latter pathway is the source of tissue-resident macrophages (TRMs) [40]. The TRMs participate in the early stages of tumor development, whereas bone marrow (BM)-derived monocytes only infiltrate the tumor microenvironment as the tumor progresses [41]. It has also been demonstrated that TRM alone is sufficient to promote tumor development at its primary site, even without the involvement of infiltrating monocytes [42]. TRMs also play a significant role in the development of metastasis. These macrophages are present in healthy tissues even before cancer cells arrival. They create favorable conditions for cancer cells to establish themselves in these locations by suppressing dendritic cells and reducing T-helper cell activation [40]. Over time, the number of macrophages derived from infiltrating monocytes can even exceed the number of TRMs, as has been observed, in both the murine mammary carcinoma model and the animal model of lung cancer [41,42]. Although BM-derived monocytes are not essential for tumor development, they can influence metastatic potential by promoting the spatial dispersion of the cellular arrangements within the tumor [42].

Based on the YUMM1.7 model, two subpopulations of macrophages were identified in the melanoma TME according to the expression of the F4/80 marker: F4/80 high and F4/80 low. It has been proven that the presence of this protein on the surface of macrophages determines their distinct phenotype. The TAMs with low expression of this marker exhibited lymphocyte antigen 6 complex, locus C (Ly6C) expression, which is characteristic of monocytes. In contrast, the absence of Ly6C on the surface of F4/80 high macrophages is also characteristic feature for resident macrophages. Despite these similarities, researchers demonstrated that nearly all TAMs originated from BM-derived monocytes in this model. It is worth noting that in other types of skin cancers, F4/80 high TAMs may partially derive from resident macrophages. The experiment also demonstrated that the number of TAMs, particularly F4/80 high macrophages, increased as the tumor progressed [43].

The macrophage subpopulation can be divided based not only on their origin but also on their interaction with the tumor. Thus, we distinguish between anti-tumor M1 macrophages, which exhibit a pro-inflammatory phenotype, and immunosuppressive M2 macrophages, which promote tumor progression [41]. To distinguish subpopulations, specific markers can be used, including: tumor necrosis factor alpha (TNF-α), IL-1β, IL-6, inducible nitric oxide synthase (iNOS), and cluster of differentiation 80 (CD80) for M1 macrophages, and arginase 1 (Arg1), chitinase-3-like protein 3 (Chi3l3), mannose receptor C-type 1 (MRC1), and cluster of differentiation 206 (CD206) for M2 macrophages [44]. M0 and M2 macrophages are quantitatively dominant in the melanoma microenvironment [44]. However, there are studies suggesting that the phenotype of most tumor-associated macrophages (TAMs) is intermediate between the M0, M1, and M2 populations. Nevertheless, it is still possible to divide them into immunosuppressive fractions (characterized by the secretion of Chi3l3) and those exhibiting pro-inflammatory activity (secreting TNF-α) [28]. TAMs in melanoma exhibit considerable phenotypic diversity. At the same time, macrophages with opposing properties may coexist within the tumor microenvironment. Furthermore, individual cells may display a variety of markers and their phenotypic profile changes significantly over the course of tumor development. Interestingly, TAMs become a much more homogeneous group in terms of the expression of key markers, after isolation from the TME. This may suggest that the acquisition of a specific phenotype is not a one-time process for macrophages but rather involves continuous interaction with tumor cells and the TME. This, therefore, represents an attractive therapeutic target [28]. In an addition to cytokines, conjunctival melanoma cells secrete large amounts of lactate, which attracts macrophages to the TME and promotes their adoption of the M2 phenotype [45], a process further supported by exosomes released by TME cells that play a significant role in driving this polarization [29]. This has been demonstrated using an inhibitor of exosome formation—GW4869, which led to a reduction in the expression of markers characteristic of M2 macrophages, such as CD206 and interleukin-4 (IL-4) [29]. Androgens also promote an immunosuppressive macrophage phenotype, primarily by inducing M2 polarization and inhibiting the TLR4/NF-κB pathway and pro-inflammatory cytokine production [46].

A significant role in recruiting macrophages to the TME may be played by TCIPA (tumor cell-induced platelet aggregation), as demonstrated in a pulmonary metastatic melanoma model. In this process, chemokine C-C motif ligand 2 (CCL2), stromal cell-derived factor 1 (SDF-1), interleukin-1 alpha (IL-1α), and IL-1β play a key role. Furthermore, increased levels of these chemokines coincided with elevated expression of their receptors on macrophages [47].

The interactions between macrophages and tumor cells, as well as with fibroblasts, are important not only in the context of their recruitment but also in their capability to increase survival and stimulate proliferation of cancer cells [28]. Interactions with melanoma cells results in an increase in the expression of chemokine C-C motif ligand 8 (CCL8) and chemokine C-C motif ligand 15 (CCL15) by tumor-associated macrophages (TAMs) and subsequent stimulation of the C-C chemokine receptor type 1 (CCR1) present, among others, on monocytes. Monocytes recruited from the peripheral blood to the tumor microenvironment (TME), form an additional layer of protection for melanoma cells. Interestingly, an experiment in which melanoma cells were introduced into the bloodstream of mice while CCL8 and CCL15 were administered subcutaneously, resulted in an increased number of tumor cell colonies embedded in the lungs, suggesting important function of those chemokines in metastasis [48]. M2-TAMs, under the influence of high lactate concentrations found in tumors, are capable of secreting vascular endothelial growth factor (VEGF), transforming growth factor beta (TGF-β), and interleukin-10 (IL-10), which promote angiogenesis [45]. A programmed death-ligand 1 (PD-L1) is an important factor responsible for the interaction between TAMs and cytotoxic T-lymphocytes, promoting tumor immune escape. It was shown that blocking this molecule with antibodies significantly reduces the mass of pulmonary melanoma metastases [47]. Cancer cells play a pivotal role in stimulation of expression of PD-1L on the surface of M2 tumor-associated macrophages (M2-TAMs) [47]. The presence of preadipocytes in the tumor microenvironment (TME) may further potentiate this process [29]. TAMs have also an ability to secrete chemokine C-C motif ligand 20 (CCL20), tumor necrosis factor (TNF), and vascular endothelial growth factor A (VEGFA), which can facilitate metastasis [48]. Studies indicate that as the tumor develops, the secretory activity of TAMs also increases [28]. Interestingly, macrophages can adopt different shapes depending on their location within the TME. TAMs located in close proximity to other cells take on an elongated shape and develop extended protrusions, significantly increasing the surface area for cell-to-cell contact. In contrast, other macrophages are generally smaller and adopt a round shape [28].

### 2.3. Tumor-Infiltrating Lymphocytes (TILs)

Lymphocytes that localize in close proximity to tumor cells and interact with them are referred to in the literature as tumor-infiltrating lymphocytes (TILs). Their presence has been demonstrated in both primary tumors and metastatic lesions [49]. They are primarily recruited from the population of circulating lymphocytes [50].

The identification of tumor-reactive T-lymphocytes has traditionally relied on the expression of markers such as CD39 and CD103. However, recent studies suggest that a specific gene expression profile, including elevated expression of programmed cell death protein 1 (PD-1), T-cell immunoreceptor with Ig and ITIM domains (TIGIT), and CXCL13, correlates better with the presence of reactive TCR clones and may serve as a more precise tool for isolating cells with potential anti-tumor activity. These findings suggest that the analysis of gene expression patterns may surpass classical surface markers in identifying functionally significant T-lymphocytes within the tumor microenvironment [51]. It is interesting to note that lymphocytes with a progenitor-like phenotype (CD39-CD69-) appear to be more significant for the durability of the tumor response compared to TILs, despite the fact that most tumor-reactive lymphocytes exhibit a more mature phenotype (CD39+) [52]. Studies have shown that a single TCR clonotype can be present in T-cells with diverse phenotypes (effector, memory, exhausted), suggesting dynamic and multi-stage interactions with the tumor microenvironment [53].

The degree of TME infiltration by lymphocytes can be also used as a separate prognostic marker (cite) in tumors. According to Clark’s method, the analyzed tumors can be classified into one of three categories: absent (when lymphocytes do not infiltrate the TME), non-brisk (when lymphocytes form focal clusters within the tumor), and brisk (when there is a diffuse infiltration of lymphocytes) [54]. The scale developed by the Melanoma Institute Australia (MIA) is also helpful in assessing the density and distribution of lymphocytes within the TME [54]. Studies have shown that a higher score obtained in these scales correlates with a milder disease course and better treatment outcomes. Specifically, a correlation has been demonstrated between TME infiltration by lymphocytes and their greater dispersion, as well as higher eight-year survival rates and the presence of metastases in sentinel lymph nodes detected in biopsies [54]. It is also suggested that the presence of CD3+ TILs at the invasive margin of the tumor is the only prognostically favorable factor, as no correlation has been found between the number and density of TILs in the tumor center and overall survival or disease-free survival [55,56]. Tumors with lymphocytic infiltration can be also classified into hot (with an inflammatory process promoted by T-cells) and cool (in which the pro-inflammatory function of lymphocytes has been suppressed) [54]. In females, tumors with abundant cytotoxic T-lymphocyte (CTL) infiltration are more common, whereas in males, tumors with low CTL infiltration tend to predominate [57]. A factor that promotes the development of the inflammatory process in the TME is the expression of PD-L1 and interferon γ [54]. It was shown that a richer lymphocytic infiltration, assessed using Clark’s method, correlates with higher PD-L1 expression within the tumor. Interestingly, its expression was significantly higher in melanomas located on the limbs and upper torso compared to acral melanoma and melanomas of the head and neck. This disparity was particularly noticeable when analyzing the amount of this protein on the lymphocytes infiltrating the tumor. The level of PD-L1 expression on both lymphocytes and tumor cells also positively correlated with the tumor’s depth according to the Breslow scale. Thus, high levels of PD-L1 have been identified as an independent prognostic factor associated with favorable outcomes in melanoma and enhanced effectiveness of immunotherapy [58].

The lymphocytes associated with the tumor immune microenvironment (TIME) are represented by all major subpopulations, including: CD20+ B-cells, CD3+CD4+ helper T-cells, CD3+CD8+ cytotoxic T-cells and the forkhead box P3-positive (FOXP3+) regulatory T-cells (Tregs) [54,59].

#### 2.3.1. B-Cells

The majority of B-lymphocytes in the tumor microenvironment cluster in TLS [60]. These are ectopic lymph node-like structures containing follicles predominantly composed of B-cells (both mature CD20+ and precursor CD79a+) [61]. The follicular structures contain a germinal center, characterized by cells expressing the activation marker CD23, which is surrounded by a mantle zone composed of IgD+ naive B-cells [62]. T-lymphocytes, in turn, are mainly located in the parafollicular areas, although a limited number of T-cells may also be present within the follicles [61]. CD21+ follicular dendritic cells are found within the follicles, while dendritic cells DC-Lamp+ (dendritic cell–lysosome-associated membrane glycoprotein) are located in the surrounding areas [61,62]. The presence of TLS is often correlated with favorable clinical outcomes in cancer patients. Studies have demonstrated that tumors rich in TLS respond more effectively to immune checkpoint inhibitors (ICI) as well as to chemotherapy. Particularly noteworthy are mature TLS enriched in B-lymphocytes (CD20+, CD23+), which have significant prognostic value [60,63]. Notably, ICB therapies can induce the formation of TLSs and enhance their functionality [63].

Beyond their aggregation in TLS, B-cells can also be found in a more dispersed manner within the tumor microenvironment (TME). However, the presence of B-lymphocytes outside TLS does not consistently correlate with improved prognosis. Dispersed tumor-infiltrating B-cells (TIL-Bs) may, in fact, contribute to tumor progression through increased expression of immunosuppressive cytokines and immune checkpoint molecules. Several subtypes of B-cells can be distinguished based on their function. Naive B-cells—T-cell leukemia/lymphoma 1A-positive (TCL1A+)—are characterized by low immunological activity due to a lack of prior antigen exposure. CXCR4+ B-cells represent a transitional population capable of differentiating into other B-cell types. Both subtypes are involved in signaling pathways associated with antigen processing and presentation; however, CXCR4+ B-cells are capable of promoting various types of immune responses by stimulating T-helper 1 (Th1), T-helper 2 (Th2), or T-helper 17 (Th17) lymphocytes. Follicular B-cells exhibit high expression of CD69, a molecule regulating the activation of both T- and B-lymphocytes [60]. Clinical observations suggest that patients with elevated CD69 expression have increased disease-free survival compared to those with low CD69 levels, which has been observed in triple-negative breast cancer [64]. It is noteworthy that CXCR4+ B-cells also express this protein. Germinal center B-cells—marker of proliferation Ki-67-positive (MKI67+)—are enriched in pathways associated with cell proliferation, DNA replication, and replication error repair, indicating their involvement in the maturation of the immune response, including somatic hypermutation within germinal centers of lymphoid structures. Plasma B-cells, representing the terminal stage of B-cell differentiation, are responsible for antibody production. These cells are characterized by high expression of PR domain zinc finger protein 1 (PRDM1), X-box-binding protein 1 (XBP1), marginal zone B- and B1-cell-specific protein (MZB1), and signal sequence receptor subunit 4 (SSR4). They also exhibit activation of pathways related to antigen processing and presentation within the endoplasmic reticulum [60].

#### 2.3.2. CD4+ T-Cells

CD4+ T-lymphocytes are primarily activated by dendritic cells (DCs) presenting tumor-associated antigens in the context of major histocompatibility complex (MHC) class II molecules [65]. However, these molecules may also be expressed on tumor cells themselves. According to earlier data, MHC class II expression was observed in over 70% of melanoma cell lines exposed to interferon-γ (IFN-γ) [66]. In a more recent study by Draghi et al. (2024), constitutive MHC II expression was detected in approximately 59% of melanoma cell lines, and this proportion increased to 95% following IFN-γ stimulation [67].

The fundamental classification of CD4+ T-lymphocytes includes the following subsets: Th1-cells, which respond to intracellular pathogens; Th2-cells, responsible for immune responses against extracellular parasites and allergens; Th17-cells, involved in defense against bacteria and fungi, but also implicated in autoimmune diseases; and regulatory T-cells (Tregs), whose primary function is to modulate immune responses and prevent autoimmunity. Tumor-associated CD4+ T-lymphocytes show unusual phenotypic plasticity, which enables them to adapt to dynamic conditions within the tissue microenvironment. Depending on the subtype, CD4+ T-cells may either promote or inhibit tumor progression [65].

A particularly important subset in the context of antitumor immunity is the Th1 (T-helper type 1) subset of CD4+ T-cells. For example, Th1 effector cells play a pivotal role in initiating and sustaining antitumor responses through the secretion of cytokines, such as interferon-γ (IFN-γ) and interleukin-2 (IL-2), which promote the activation and proliferation of CD8+ T-lymphocytes, as well as other immune effector cells [65]. In the context of the aforementioned cytokines, the functional differentiation of memory CD4+ T-cells is also of particular importance. These cells are divided into two main subsets: central memory T-cells, which are characterized by the expression of CCR7 and CD62L, enabling their recirculation through lymph nodes. They primarily secrete IL-2. In contrast, effector memory T-cells, which lack CCR7, are capable of rapidly producing both IL-2 and IFN-γ, for example, in response to infection [68]. Moreover, Th1-cells are essential for the effective priming of dendritic cells (DCs) for antigen presentation, which is critical for the robust activation of CD8+ T-cells and the subsequent development of their effector functions and immunological memory. IFN-γ production by Th1-cells can also induce interleukin-12 (IL-12) secretion by DCs, thereby amplifying the Th1 response via a positive feedback loop. In addition, the synergistic action of IFN-γ and tumor necrosis factor alpha (TNF-α) can promote tumor cell senescence, inhibit tumor cell proliferation, and contribute to the remodeling of the tumor vasculature. These effects collectively reduce tumor vascularization and support the elimination of malignant cells [65].

The primary function of Th2-cells is their involvement in the elimination of extracellular pathogens and allergic responses. In the context of cancer, it has been suggested that Th2-lymphocytes may exhibit both tumor-promoting and antitumor activities. Th2-cells can facilitate angiogenesis and suppress Th1 responses, thereby supporting tumor immune evasion. Through IL-4, they can promote the polarization of macrophages toward a phenotype that favors metastasis. Interestingly, although M2 macrophages are generally associated with protumor activity, their secretion of arginase can inhibit tumor progression. Thus, their recruitment to the tumor microenvironment by Th2-cells may also have beneficial effects [69]. A recent study demonstrated that Th2-lymphocytes exert significant antitumor effects in murine allograft models of colorectal and pancreatic cancer. Administration of Th2-cells led to a marked reduction in tumor growth—by as much as 50% following a single injection—with repeated administrations producing an even more pronounced therapeutic effect. These antitumor outcomes were associated with a substantial increase in eosinophil and macrophage infiltration within the tumor microenvironment, suggesting a reprogramming of the innate immune response toward an antitumor phenotype [70]. Cytokines such as IL-5, IFN-γ, and TNF significantly support the antitumor functions of eosinophils. This activity may manifest through the secretion of cytotoxic molecules like MBP and granzymes, as well as by stimulating NK-cells and CD8+ T-cells. However, it should be noted that eosinophils may, depending on the tumor microenvironment, exhibit pro-tumor properties—for example, by recruiting Treg cells [71]. Consistent with these observations, Th2 cell therapy has been shown to enhance cytotoxic and apoptotic pathways within tumors, including upregulation of MBP, MPO, Nos2, granzyme B, perforin, and Fas/FasL signaling. Both Th2-cells and eosinophils were directly implicated in promoting tumor cell apoptosis, as confirmed by in vitro experiments and elevated levels of soluble GZMB and FAS. A key mediator of this effect was IL-5: its increase following Th2 therapy correlated with tumor inhibition and eosinophil infiltration, although recombinant IL-5 alone induced a milder response compared to full Th2-cell administration [70].

Th17-lymphocytes play a significant role in the process of skin carcinogenesis, by secretion of IL-17, which in turn stimulates the expression of chemokines such as C-X-C motif chemokine ligand 1 (CXCL1) and C-X-C motif chemokine ligand 2 (CXCL2). Those chemokines promote the recruitment of neutrophils and macrophages and increase the levels of pro-inflammatory cytokines including tumor necrosis factor alpha (TNF-α) and interleukin-1β (IL-1β). Additionally, IL-17 induces the expression of anti-apoptotic proteins (e.g., Bcl-XL, Survivin) and cell cycle regulators (e.g., Cyclin D1), thereby supporting epithelial cell proliferation. In the context of melanoma, Th17-cells may contribute to tumor angiogenesis through the regulation of vascular endothelial growth factor (VEGF) and matrix metalloproteinase 9 (MMP9). However, in the presence of interferon gamma (IFN-γ), Th17-cells can also exhibit antitumor potential by facilitating the activation of CD8+ T-lymphocytes via dendritic cell priming [65].

Although cytotoxic properties are primarily associated with CD8+ T-cells, it is important to mention a subset of CD4+ lymphocytes (cytotoxic CD4+ T-lymphocytes—CD4+ CTLs) that also possess the ability to directly kill cancer cells, albeit with MHC class II restriction. These cells require stronger stimulation than CD8+ T-cells to achieve their cytotoxic function. Transcription factors promoting the development of cytotoxicity in these cells include eomesodermin (EOMES) (which is strongly induced by interleukin-2, IL-2), T-box transcription factor (T-bet, TBET), and runt-related transcription factor 3 (RUNX3). Depending on the conditions, CD4+ lymphocytes exhibiting cytotoxic properties can arise from Th0-, Th1-, or Th2-cells. The direct cytotoxic activity of these CD4+ T-cells is dependent on the secretion of IFN-γ, as well as the release of granzymes and perforin, but it is independent of the Fas and TNF-related apoptosis-inducing ligand (TRAIL) pathways. Studies have shown that stimulation via OX40 (CD134), administration of anti-cytotoxic T-lymphocyte-associated protein 4 (anti-CTLA-4) antibodies, or chemotherapy with cyclophosphamide can enhance the cytotoxic potential of these cells. In addition to granzymes and perforin, cytotoxic CD4+ cells express granulysin (GNLY) and natural killer cell granule protein 7 (NKG7), while their surface also displays lysosomal-associated membrane protein 1 (LAMP-1, CD107a), a marker of degranulation [72]. An intriguing phenomenon is that the loss of IFN-γ signaling (e.g., due to mutations in the JAK1 gene) leads to the inability to induce MHC class II expression on tumor cells, thus preventing their recognition and elimination by CD4+ CTLs. However, tumors that exhibit constitutive MHC class II expression can be still recognized and eliminated by these cells independently of IFN-γ signaling [67].

#### 2.3.3. CD8+ T-Cells

CD8+ lymphocytes exhibit cytotoxic properties, promoting the removal of cancer cells through both indirect mechanisms (via interferon γ and TNF signaling) and direct mechanisms (through the secretion of exosomes containing granzymes and perforins) [54]. It should be emphasized that cytotoxic T CD8+ cells are considered the most powerful effectors of the anticancer response among the immune repertoire being the backbone of cancer immunotherapy with immune checkpoint inhibitors anti-CTLA-4 (cytotoxic T-lymphocyte associated protein 4) or anti-PD-1 (programmed cell death protein 1), including melanoma [73,74]. Human leukocyte antigen class I (HLA-I) plays a crucial role in the activation of cytotoxic T-lymphocytes. Through its interaction with the T-cell receptor (TCR) present on lymphocytes, the recognition of tumor cells or virus-infected cells is made possible. However, the stimulation of T-cells via this pathway is often too weak in the case of tumor cells, as TCRs are more adapted to recognizing antigens derived from pathogens. There is, however, a type of cytotoxic T-lymphocyte response known as multipronged, which is based on responding to multiple tumor-associated antigens by recognizing various epitopes. This capability was observed in the T-cell clone MEL8, derived from the infusion of tumor-infiltrating lymphocytes (TILs) from a patient with stage IV melanoma, in which complete remission occurred. In these lymphocytes, the presence of additional antigens can significantly enhance their activation, although a single antigen is sufficient to trigger the cytotoxic effect. Studies suggest that the underlying mechanism for this ability is molecular mimicry, where antigens exhibiting structural similarities in fragments crucial for TCR interaction are recognized [75]. A β2 microglobulin (B2M) deficiency due to mutation results in reduced level of MHC I on the surface of tumor cells. The acquisition of mutations in this gene corelate with melanoma progression in patients who had previously experienced remission after immunotherapy. This suggests that the proper functioning of MHC I, as well as B2M is crucial in the context of immunotherapies, as it is essential for the recognition of antigens by CD8+ lymphocytes. On the other hand, natural killer (NK) cells may offer a potential pathway to mitigate this phenomenon due to the lack of inhibitory signals from absent MHC I molecules. This possibility was demonstrated in a mouse model, where tumors in mice with reduced NK-cell levels exhibited a higher number of tumor cells with MHC I deficiency [76].

#### 2.3.4. Tregs

Tregs are a subset of CD4+ T-lymphocytes characterized by the expression FOXP3 (a transcription factor that regulates their suppressive function) and CD25 (the alpha chain of the IL-2 receptor, which serves as a marker of their activation). An important marker in the context of these cells is CD127, as a low level of this receptor correlates with the immunosuppressive activity of Tregs [59]. In melanoma, Tregs are key mediators of immune tolerance, suppressing the anti-tumor immune response. These cells are found in peripheral blood, sentinel lymph nodes, tumor-infiltrating lymphocytes, and in peripheral skin, where they are particularly present near blood vessels and dendritic cells. As the disease progresses, the number of this subpopulation also increases [59,77]. Testosterone promotes the expansion and function of Treg cells, partly by inducing FoxP3 expression via the androgen receptor; a deficiency of this hormone leads to reduced Treg number and activity [46].

Tregs can generate adenosine through the enzymes CD39 and CD73. Adenosine inhibits the activity of dendritic cells (DCs) and exerts an immunosuppressive effect on effector T-lymphocytes. This mechanism is particularly pronounced in melanoma tumors [59]. In the context of CD73, it is worth noting that in a murine model of chemically induced mammary gland tumorigenesis, CD73 gene knockout (CD73 KO) was shown to delay the development of HR/PR-negative tumors by reprogramming lipid metabolism. This was accompanied by increased expression of genes involved in fatty acid biosynthesis and oxidation, as well as elevated oxidative stress and mutational burden, which may contribute to genomic instability and a delayed tumor onset [78].

Tregs can be divided into four subtypes: nTregs (natural Tregs) CD39+CD73-, which are responsible for regulating tolerance to self-antigens; iTregs (induced Tregs) CD39+CD73+, which represent the most immunosuppressive subpopulation in melanoma, responsible for inhibiting the immune response, and their highest presence has been detected in tumor infiltration; oTregs (other Tregs) CD39-CD73+, which may be generated by tumors as an additional mechanism of immune evasion; and xTregs (non-classical Tregs) CD39-CD73-, whose immunosuppressive action is based on the secretion of TGF-β and IL-10, rather than adenosine [59].

### 2.4. Dendritic Cells (DCs)

Conventional dendritic cells (cDCs) are cells capable of antigen presentation, and their influence on immunity primarily depends on their diverse interactions with T-lymphocytes. Their ability to stimulate these cells is the highest among all immune cells. DCs are present in the tumor microenvironment (TME), although they constitute only a small fraction of immune cells, and they can also migrate from the TME to lymph nodes. In both locations, they can present tumor antigens to T-cells [79]. Estrogen signaling via ERα modulates dendritic cell development and function, enhancing cytokine production and T-cell activation, and thus contributes to sex-based differences in immune responses [80].

So far, the best-characterized group of dendritic cells is conventional DCs. They can be divided into two main subtypes: cDC1 (dependent on the transcription factors basic leucine zipper ATF-like transcription factor 3 (BATF3), interferon regulatory factor 8 (IRF8), and inhibitor of DNA binding 2 (ID2) and cDC2, dependent on the transcription factors v-rel avian reticuloendotheliosis viral oncogene homolog B (RELB), interferon regulatory factor 4 (IRF4), zinc finger E-box-binding homeobox 2 (ZEB2), inhibitor of DNA binding 2 (ID2), and Notch homolog 2 (Notch2) [79,81].

cDC1 cells are primarily recruited to the TME by natural killer (NK) cells, mainly under the influence of the chemokines X-C motif chemokine ligand 1 (XCL1) and C-C motif chemokine ligand 5 (CCL5). A crucial growth factor required for dendritic cell survival is FMS-like tyrosine kinase 3 ligand (FLT3L), which is also provided by NK-cells. Characteristic markers of cDC1 include the chemokine receptor X-C motif chemokine receptor 1 (XCR1), the C-type lectin receptor dendritic cell NK lectin group receptor-1/C-type lectin domain family 9 member A (DNGR-1/CLEC9A), the integrin alpha E (CD103), and the transmembrane protein blood dendritic cell antigen 3/cluster of differentiation 141 (BDCA3/CD141) [79]. cDC1 cells play a key role in stimulating immune control over tumors. They are capable of capturing antigens from dead cell debris and cross-presenting them to naïve T-lymphocytes in lymph nodes, stimulating their differentiation into CD8+ T-cells. Additionally, cDC1 cells remaining in the TME secrete chemokines that promote the influx of these cells into the tumor microenvironment, where further antigen presentation leads to additional stimulation [79,82].

Until a few years ago, our knowledge of cDC2 cells was limited, primarily due to the lack of specific markers as CD11b and CD172a could not be considered as selective markers [79]. Currently, we have a broad range of molecules that allow the identification of this subpopulation, significantly improving our understanding of its role in immune processes. In addition to the aforementioned markers, cDC2 cells are characterized by blood dendritic cell antigen 1/cluster of differentiation 1c (BDCA1/CD1c), cluster of differentiation 11c (CD11c), CD5, high-affinity immunoglobulin epsilon receptor subunit alpha (FCεR1), C-C motif chemokine receptor 2 (CCR2), and B- and T-lymphocyte attenuator (BTLA) [81]. It is known that cDC2 cells exhibit a higher potential for stimulating CD4+ T-cells compared to subtype 1 [82].

The described interactions above pertain to migratory dendritic cells (DCs CD103+), whereas there is also a lymphoid subpopulation (DCs CD8α+), which resides in lymph nodes. Cells belonging to this population generally exhibit lower MHC II expression compared to migratory DCs; however, it is important to note that the expression of this protein can increase upon activation. Resident DCs are limited to processing antigens delivered by migratory DCs [79,82].

Langerhans cells represent a particularly important DC subtype in the context of melanoma, as they are the only subpopulation residing in the epidermis. Moreover, they constitute the most abundant DC population in sentinel lymph nodes of melanoma patients. They migrate in response to tissue damage, which triggers their activation and acquisition of antigen-presenting capabilities. Under physiological conditions, LCs express interferon alpha (IFN-α) and interferon beta (IFN-β), as well as the pro-inflammatory cytokines IL-6 and interleukin-8 (IL-8), and TNF-α. It has been demonstrated that tumor cells in the sentinel lymph node (SLN) secrete immunosuppressive cytokines (e.g., IL-10, TGF-β), which impair Langerhans cell function by downregulating the expression of costimulatory molecules CD80/CD86 and maturation markers (CD83). Consequently, LCs remain in an immature state, promoting tolerance to tumor antigens and facilitating early metastasis. Additionally, in a subset of immature LCs (CD83−) in the SLN, indoleamine 2,3-dioxygenase 1 (IDO1) expression is increased, which may enhance immunosuppression and inhibit T-cell proliferation. This phenomenon represents a potential therapeutic target [83].

Another dendritic cell subtype is plasmacytoid DCs (pDCs), which, interestingly, can exhibit cytotoxic activity via TRAIL (TNF-related apoptosis-inducing ligand) and granzyme B as it was shown in murine models of breast cancer and melanoma. TRAIL is a pro-apoptotic molecule present in the cytoplasm that, upon activation of dendritic cell via toll-like receptor 7 (TLR7) and toll-like receptor 9 (TLR9) receptors, can be exposed on the cell surface and induce tumor cell death through interaction with death receptors: TRAIL-R1 (DR4) and TRAIL-R2 (DR5) [84]. In human cancers, such as basal cell carcinoma, it has been demonstrated that pDCs stimulated via TLR7 (e.g., with imiquimod, CpG—cytosine-phosphate-guanine, IFN-α) acquired the ability to induce tumor cell apoptosis in a TRAIL-dependent manner. In skin cancers such as melanoma, pDCs are recruited to the TME via chemokine C-C motif ligand 20 (CCL20). pDCs can promote antitumor immunity by producing IFN-α, activating NK-cells, and presenting antigens to CD8+ T-lymphocytes. Nevertheless, in most cancers, including melanoma, the presence of pDCs is negatively correlated with patient prognosis. This may be due to the fact that in the TME, pDCs frequently acquire a tolerogenic phenotype, which is associated with the presence of immunosuppressive cytokines (e.g., TGF-β, IL-10), tumor metabolites, and interactions with inhibitory receptors (e.g., blood dendritic cell antigen 2—BDCA-2, immunoglobulin-like transcript 7—ILT7). Tolerogenic pDCs often express immunosuppressive molecules such as inducible T-cell co-stimulator ligand (ICOS-L), indoleamine 2,3-dioxygenase (IDO), and programmed death-ligand 1 (PD-L1), promoting Treg expansion and suppressing antitumor responses. It is also worth noting that while granzyme B secreted by DCs can contribute to tumor cell apoptosis, it may also exert suppressive effects on T-lymphocytes. Thus, pDCs are associated with multiple potential targets for immunotherapy [84].

Relatively recently, dendritic cells type 3 (DC3) has been identified as a separate subpopulation of DC. Phenotypically, they resemble cDC2 CD301b+ but they do not arise from the same common dendritic progenitor (CDP) but rather from the Ly6C+ monocyte-dendritic progenitor (MDP) fraction. Fc gamma receptor IIB/III (FcγRIIB/III) is a key marker distinguishing DC3s from cDC2s. Additionally, the presence of this receptor suggests that they may be more sensitive to immunoglobulin-mediated signaling; however, further research is needed to determine how this affects their immunological function. DC3s also express monocyte-associated markers, indicating a mixed phenotype. Finally, these cells are capable of inducing a Th17-type immune response [85].

In the context of DCs, it is important to mention clusterin, a glycoprotein that plays a key role in protecting these cells from oxidative stress [86]. Experimental studies involving clusterin gene silencing have shown that DCs undergo apoptosis more rapidly and exhibit reduced capacity to activate T-lymphocytes. However, this protein is not present in all DCs but is specifically expressed by mregDCs (mature regulatory dendritic cells), a subpopulation found in the TME. Characteristic markers of this population include lysosomal-associated membrane protein 3 (LAMP3), C-C motif chemokine receptor 7 (CCR7), cluster of differentiation 83 (CD83), baculoviral IAP repeat-containing 3 (BIRC3), and myristoylated alanine-rich C-kinase substrate-like protein 1 (MARCKSL1). Interactions with T-lymphocytes may contribute to the differentiation of DCs into the mregDC phenotype. This subpopulation exhibits an increased ability to capture antigens but a reduced capacity to activate antitumor responses due to high expression of immunoregulatory and inhibitory molecules such as CD200 and PD-L1. It is worth noting that while clusterin supports DC survival and thus enhances antitumor immunity, it also facilitates tumor cell survival through its anti-apoptotic effects. Furthermore, reports suggesting that mregDCs may suppress immune responses against tumors provide a rationale for further research on this subpopulation, underlining the role of clusterin [82,87]. Interleukin-27 (IL-27) is another highly relevant protein in the context of DC function. It is primarily produced by dendritic cells capable of activating T-lymphocytes in lymph nodes. Blocking IL-27 (using antibodies against its p28 subunit) reduced the number of CD4+ T-lymphocytes in the tumor microenvironment (TME), likely due to decreased expression of C-X-C chemokine receptor 3 (CXCR3) on CD4+ T-cells, a receptor responsible for their migration to tumors. IL-27 is a key factor promoting IFN-γ production by both CD4+ and CD8+ T-cells. In a murine melanoma model, administration of IL-27-neutralizing antibodies significantly reduced IFN-γ production by T-lymphocytes in both lymph nodes and the tumor. A blockade of IL-27 activity or knockdown of coding the gene, was shown to stimulate tumor development, providing strong evidence that IL-27 is a crucial factor in supporting antitumor immunity [82]. Experimental studies have demonstrated that immune checkpoint inhibitors (CTLA-4 and PD-1 blockade) enhance dendritic cell interactions with T-lymphocytes. Treatment of mice with anti-CTLA-4 antibodies increased the number of DCs directly interacting with T-cells via the CD40-CD40L pathway. An accumulation of DC was observed both in the TME and in tumor-draining lymph nodes, with this effect being particularly pronounced for cDC2s. Additionally, an upregulation of costimulatory molecules such as CD40, CD86, and CD80 was observed on DCs, strengthening their interactions with T-lymphocytes. The PD-1 blockade had a lesser effect than the CTLA-4 blockade, but still resulted in an increase in number of active DCs in the TME. Notably, mregDCs exhibited a weaker response to therapy compared to cDCs1 and cDCs2. These observed patterns indicate a crucial role for dendritic cells as mediators of the immune response induced by immune checkpoint blockade [82].

The major subsets of melanoma microenvironment cells and their characteristic features are summarized in Table 1.

## 3. Therapeutic Implications and Future Perspectives

The treatment of melanoma has undergone a significant evolution—from the dominance of surgical methods and non-specific immunomodulatory approaches (such as BCG vaccine or LAK cells with interleukin-2), through the FDA approval of dacarbazine in 1976 as chemotherapy for advanced disease and subsequent registrations of high-dose interferon and interleukin-2, to the groundbreaking therapies introduced in 2011—ipilimumab and vemurafenib [88]. New melanoma therapies, based on immunotherapy and targeted treatment, have significantly improved survival in patients with advanced-stage disease, revolutionizing clinical approaches and prognosis. The most recent immunotherapy regimens demonstrate even greater efficacy [89,90,91,92,93]. Despite the high efficacy of immune checkpoint inhibitors in some melanoma patients, up to 55% exhibit primary resistance to anti-PD-1 therapy, and an additional 25% develop secondary resistance within two years [94]. The mechanisms of this resistance include, among others, loss of MHC expression, mutations in the B2M gene, tumor cell de-differentiation, and disruptions in signaling pathways such as PTEN [94,95]. Both preclinical and clinical studies highlight the need for novel therapeutic strategies due to the limited persistence of treatment responses and the heterogeneous landscape of immunoresistance in melanoma [96,97]. Currently, neoadjuvant immunotherapy is recommended for the treatment of resectable stage IIIB–IV melanoma, primarily involving the use of a PD-1 inhibitor, such as pembrolizumab [98]. In adjuvant treatment for patients with stage IIB–IV melanoma, immune checkpoint inhibitors (nivolumab, pembrolizumab) are used, and in cases of BRAF mutation, targeted therapy with BRAF inhibitors combined with MEK inhibitors is administered [98,99,100]. In advanced stages of the disease (unresectable stage III–IV or metastatic), first-line treatment consists of combination immunotherapy regimens (nivolumab with ipilimumab or relatlimab). In the presence of a BRAF mutation, targeted therapy is also an option, although immunotherapy demonstrates greater durability of response and superiority in overall survival [98,100,101]. In selected cases, cellular therapy (tumor-infiltrating lymphocytes, TILs) or oncolytic viruses (e.g., T-VEC) are also employed [102,103]. In the multicenter phase II study (C-144-01) investigating lifileucel therapy, a one-time autologous tumor-infiltrating lymphocyte cell treatment, in patients with advanced melanoma resistant to PD-1 inhibitors and BRAF/MEK-targeted therapy, the overall response rate was 31.4%, and the median duration of response was not reached (≥27.6 months), with 41.7% of responses lasting ≥18 months. These results underscore the potential of TILs as an integral component of modern treatment strategies for advanced melanoma [104]. Melanoma, however, establishes a strongly immunosuppressive microenvironment through the recruitment of cells such as regulatory T-cells (Tregs), tumor-associated macrophages (TAMs), and mature regulatory dendritic cells (mregDCs), which significantly limits the efficacy of immunotherapies [105,106,107].

Increasing evidence suggests that the composition of the gut microbiome may influence the efficacy of immunotherapy, particularly immune checkpoint inhibitors [108]. Routy et al. conducted a study on germ-free mice in which fecal microbiota transplantation (FMT) from patients responding to anti-PD-1 therapy enhanced treatment efficacy, in contrast to FMT from non-responding patients [108]. It was observed that the presence of Akkermansia muciniphila correlated with clinical response, and its oral supplementation in mice restored the effectiveness of PD-1 blockade in an interleukin-12-dependent manner by promoting the recruitment of CD4+ CCR9+ CXCR3+ T-lymphocytes to the tumor [108]. The association between enhanced efficacy of immunotherapies and the gut microbiota may also result from the stimulation of proinflammatory cytokine production and the secretion of tail length tape measure protein and inosine by bacteria, which augment T-cell responses [109,110]. Interestingly, Matson et al. demonstrated in mice colonized with microbiota from patients responding to immunotherapy a higher presence of tumor antigen-specific CD8+ T-cells within the tumor microenvironment [111]. Among commensal bacteria whose presence correlates with improved response to immune checkpoint inhibitors (ICI), in addition to the aforementioned *Akkermansia muciniphila*, are *Bifidobacterium longum*, *Collinsella aerofaciens*, *Enterococcus faecium*, *Faecalibacterium prausnitzii*, *Bifidobacterium pseudolongum*, *Lactobacillus johnsonii,* and *Olsenella* [109,110,111,112]. A review of clinical studies by Oh et al. suggests that greater gut microbiome diversity may positively influence the response to immune checkpoint inhibitor (ICI) therapy. The researchers also noted that two of the analyzed studies demonstrated that fecal microbiota transplantation (FMT) from patients responding to immunotherapy improved treatment response in patients resistant to anti-PD-1 therapy, without increasing toxicity [112]. FMT shows potential as an adjunct therapy to immunotherapy, chemotherapy, and radiotherapy; however, its clinical application requires further research and standardization of procedures. Meanwhile, probiotics, due to their immunomodulatory properties and beneficial effects on the tumor microenvironment, may enhance the efficacy of immune checkpoint inhibitor (ICI) therapy by promoting the growth of favorable gut bacteria [113].

A potential enhancement of immune checkpoint inhibitor therapy is represented by the fusion of IL-21 with an anti-PD-1 antibody (PD-1Ab21), which enables targeted delivery of IL-21 to tumor-reactive T-lymphocytes. This promotes the generation and expansion of memory T-cells with stem cell-like properties (TSCM), resulting in a stronger antitumor response than the standard combination of PD-1 blockade and IL-21 administration [114].

Analysis of clinical studies indicates a variable complete response rate (CRR) in tumor-infiltrating lymphocyte (TIL) therapy, reflecting the unstable efficacy of this approach [115]. The causes of this variability likely include factors such as the quality of the TILs themselves, their capacity for in vivo expansion, functional exhaustion and dysfunction of lymphocytes, as well as immunosuppression within the tumor microenvironment. Consequently, further refinement of TIL therapy and overcoming its associated challenges are essential [115]. A potential solution to some of these challenges may lie in the use of TIL-derived induced pluripotent stem cells (iPSCs), from which high-quality TILs could be obtained at an earlier developmental stage. These TILs could also exhibit greater diversity in terms of T-cell receptor (TCR) repertoire, enabling the recognition of rare tumor antigens [116,117]. Preclinical studies have demonstrated that the efficacy of TIL-based therapy can be significantly enhanced through lymphodepletion, which reduces the number of regulatory T-cells, stimulates the secretion of homeostatic cytokines (IL-7, IL-15) that support TIL expansion, and activates antigen-presenting cells via the translocation of ligands for Toll-like receptors, such as lipopolysaccharides (LPSs) [102].

The combination of TIL therapy with oncolytic viruses (OVs) significantly enhances the efficacy of treatment for solid tumors [115]. OVs induce the recruitment and accumulation of TILs with higher tumor specificity, featuring a reduced proportion of exhausted and regulatory cells [115,118]. Simultaneously, oncolytic viruses (OVs) modulate the tumor microenvironment by inducing the secretion of proinflammatory cytokines—such as IFN-γ, TNF-α, IL-2, and CXCL10—and promoting the activation of TILs [119]. Some oncolytic viruses, such as OV-OX40L/IL12 based on herpes simplex virus type 1 (HSV-1), are capable of reprogramming tumor cells into artificial antigen-presenting cells (aAPCs). This enables the provision of local signals necessary for enhanced activation of tumor-infiltrating lymphocytes (TILs), thereby increasing their ability to recognize and destroy cancer cells [120]. Treatment with a combination of Ibrutinib and Rapamycin, to block interleukin-2-inducible kinase (ITK) and mTOR pathways, respectively, leads to a reduction in exhaustion markers such as PD-1, while additionally activating pathways related to DNA repair, adhesion, and immune cell migration. This renders this therapeutic approach a potentially effective adjunct to TIL-based therapy [121]. The combination of a 4-1BB (CD137) ligand fused with fibroblast-activating protein (FAP–4-1BBL) and T-cell receptor (TCR) activation significantly enhanced the activation and effector functions of tumor-infiltrating lymphocytes (TILs), including the induction of IL-13 secretion and activation of the STAT6 signaling pathway [122]. Pentoxifylline (PTXF), a xanthine derivative with antitumor properties, exerts a beneficial effect on the function of TILs by increasing the proportion of cytotoxic lymphocytes, reducing the number of Tregs, decreasing TGF-β levels, and enhancing the expression of T-bet and production of IFN-γ, thereby promoting antitumor responses [123]. Modification of TILs using CRISPR/Cas9 to knock out TGFBR2 enables the generation of T-cells that are more resistant to immunosuppression and exhibit enhanced effector functions, representing a promising therapeutic strategy [124]. Simultaneously, innovative strategies combining features of TIL and CAR-T-cells are being developed. Preclinical studies have demonstrated that TILs enriched with CAR targeting HER2 effectively eliminate melanoma cells both in vitro and in vivo, findings that have also been confirmed in canine models resistant to checkpoint blockade [125]. The first clinical trial using GD2-CAR-T therapy in patients with metastatic melanoma demonstrated that the treatment was well-tolerated, although clinical efficacy was limited [126]. According to recent analyses, the main barriers limiting the efficacy of CAR-T therapy in solid tumors are antigen heterogeneity, antigen loss, limited tumor penetration, and an immunosuppressive microenvironment. This underscores the rationale for combining CAR-T with TILs and other immunotherapies to enhance treatment effectiveness [127,128,129,130].

Personalized neoantigen vaccines represent a promising strategy in melanoma therapy, demonstrating the ability to induce and enhance antitumor T-cell responses. In a clinical trial involving melanoma patients treated with the NeoVax vaccine, long-term persistence of neoantigen-specific memory T-cells and diversification of T-cell receptor (TCR) clones were observed, indicating a durable and dynamic immune response [131]. Similarly, the NEO-PV-01 vaccine in combination with a PD-1 inhibitor induced cytotoxic responses of CD4+ and CD8+ lymphocytes in all patients, which were capable of migrating to and destroying the tumor [132]. Additionally, epitope spreading was observed [132]. Moreover, a phase 2b study demonstrated that combination therapy with mRNA-4157 (V940) and pembrolizumab in the adjuvant treatment of resected melanoma at high risk of recurrence prolonged relapse-free survival compared to pembrolizumab monotherapy, with an acceptable safety profile [133]. Collectively, these data indicate that personalized neoantigen vaccines may serve as an effective complement to current immunotherapy strategies in melanoma [131,132,133].

**Figure 4:**
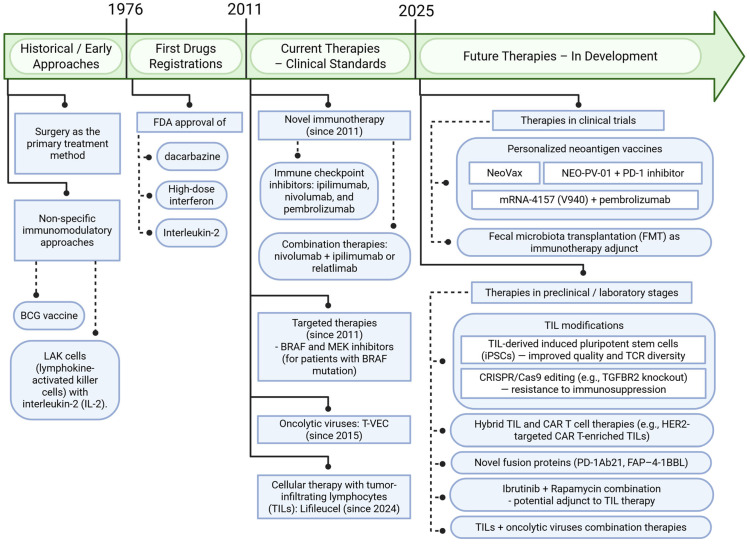
Advances in melanoma therapy: past, present, and future with a focus on the tumor microenvironment (TME). Created in BioRender. Sikorski, H. (2025) https://app.biorender.com/illustrations/6864344d6f7b03928f4b99dd (accessed on 11 July 2025).

The summary presented in Figure 4 illustrates the changes and main directions in the development of melanoma treatment, including both conventional therapies and future concepts based on the tumor microenvironment (TME).

## 4. Conclusions

The tumor microenvironment (TME) plays a central role in the initiation, progression, and therapeutic response of melanoma cells. It constitutes a dynamic network of cellular and non-cellular components that interact with one another and modulate disease course. Of primary importance are cancer-associated fibroblasts (CAFs), which, through the secretion of cytokines, chemokines, and extracellular matrix-remodeling enzymes, support tumor growth and contribute to resistance to treatment—particularly by forming physical barriers and promoting immunosuppression. Tumor-associated macrophages (TAMs) are also critical; their phenotypes shift over tumor progression and the predominance of the M2 form is linked to angiogenesis, suppression of immune responses, and facilitation of metastasis. Tumor-infiltrating lymphocytes (TILs)—including CD8+ and CD4+ T-cells, regulatory T-cells (Tregs), and B-cells (CD20+)—constitute a key element of anti-tumor immune surveillance. The abundance, spatial distribution, and functional status of TILs strongly correlate with the prognosis and the effectiveness of immunotherapy. Specific TIL subpopulations may exhibit either cytotoxic and immunostimulatory functions or suppressive properties, especially Tregs, making them not only biomarkers, but also potential therapeutic targets. Moreover, the presence of progenitor-phenotype TILs, enhance therapeutic responses and the presence of tertiary lymphoid structures (TLS) rich in B-cells correlates with improved outcomes. Dendritic cells, although less abundant, play an essential role in the activation of T-cells; yet, under the influence of TME-derived factors, they may adopt a tolerogenic phenotype that undermines immunotherapeutic efficacy. The dynamic development of melanoma therapies—including immunotherapy and targeted treatments—has significantly improved outcomes for patients with advanced disease. The introduction of immune checkpoint inhibitors such as anti-PD-1 and anti-CTLA-4 antibodies marked a major breakthrough, although more than half of patients still exhibit primary or acquired resistance. Key mechanisms of resistance include loss of MHC expression, B2M gene mutations, tumor cell de-differentiation, and disruptions in signaling pathways such as PTEN. To overcome these challenges, novel approaches are being explored, including tumor-infiltrating lymphocyte (TIL) therapy, personalized neoantigen vaccines, oncolytic viruses, and CRISPR/Cas9-based cell modifications. Increasing evidence suggests that the gut microbiome may influence the efficacy of immunotherapy, opening new avenues for adjunct strategies such as fecal microbiota transplantation (FMT) and probiotics. A growing number of preclinical and early clinical studies focus on modulating the tumor microenvironment to enhance immune responses and reverse local immunosuppression. Although not yet widely implemented in clinical practice, these tumor microenvironment-targeted strategies hold significant promise for the future of personalized melanoma treatment.

## Figures and Tables

**Table 1:** Summary of main subpopulations in melanoma tumor microenvironment (TME).

Analyzed Feature	CAFs	Refs.	TAMs	Ref.	TILs	Refs.	DCs	Refs.
Origin	Resident fibroblasts, BM-MSCs, EMT, EndMT, MMT, adipocytes, pericytes	[25]	TRMs—derived from yolk sac and fetal liver BM-derived monocytes—from myeloid progenitors in the bone marrow	[40,41,42,43]	Recruited from the population of circulating lymphocytes	[50]	CDP-derived, MDP-derived, lymph node-derived, epidermis-derived	[79,81,82,83,85]
Activation mechanisms	Exosomes secreted by cancer cells, IL-1β, IL-6, IL-8, TGF-β, PDGF, FGF-2, miR-211/155	[34]	Lactate, exosomes secreted by cancer cells, TCIPA (CCL2, IL-1α/β, SDF-1), CCL8, CCL15	[28,29,47,48]	IFN-γ/IL-2/IL-4/IL-6/IL-21/TGF-β, antigen presentation by DC, interaction with MHC-I/MHC-II	[54,60,65,72]	XCL1, CCL5, FLT3L, TLR7, TLR9, tissue damage, contact with T-cells, CD40–CD40L interaction, CTLA-4/PD-1 blockade, IL-27	[79,82,83,84,87]
Markers	mCAFs: COL1A1, COL1A2, COL3A1, LUM, POSTN, TNC; iCAFs: MMP1, MMP3, IL6, CXCL8, IDO1; myoCAFs: ACTA2, COL1A1, RGS5, KCNJ8, MCAM; other markers: FSP-1, vimentin, FAP, podoplanin, PDGFR α/β, decorin, osteonectin, desmin, CD90/THY1, NG2	[34,36]	M1: TNF-α, IL-1β, IL-6, iNOS, CD80; M2: Arg1, Chi3l3, MRC1, CD206	[44]	T: CD3; Th: CD4; Treg: FOXP3, CD25; cytotoxic: CD4, EOMES, TBET, RUNX3/CD8 degranulation: GNLY, NKG7, CD107a; tumor-reactive profile: CD39/CD103, PD-1, TIGIT, CXCL13; B: CD20, CD23/CD69/CXCR4/TCL1A/MKI67 (subtype dependent); plasma cells: PRDM1, XBP1, MZB1, SSR4	[51,54,59,60,65,67,72]	cDC1: XCR1, DNGR-1, CLEC9A, CD103, BDCA3, CD141 cDC2: BDCA1, CD1c, CD11c, CD5, FCεR1, CCR2, BTLA LCs: CD83, IDO1 pDCs: BDCA-2, ILT7, TRAIL, Granzyme B, ICOS-L, IDO, PD-L1 mregDCs: LAMP3, CCR7, CD83, BIRC3, MARCKSL1, PD-L1, CD200, Clusterin DC3s: FcγRIIB/III	[79,81,82,83,84,85,87]
Functions	ECM remodeling, physical barrier for immune cells, production of immunosuppressive cytokines, promotion of cancer cell migration and invasiveness	[5,34,36]	Phagocytosis and antigen presentation, promotion of angiogenesis (VEGF, TGF-β, and IL-10), potential support of primary tumor development (TRMs) and metastasis (M2), facilitation of immunosuppression (PD-L1)	[40,42,45,47,48]	B: a crucial component of TLS, antigen processing, antibody production; T: cytotoxicity (cytotoxic CD4+/CD8+), recruitment and stimulation of immune cells (Th), induction of tolerance, adenosine secretion(Treg)	[54,59,60,65,70,72]	Antigen uptake, presentation, cross-presentation, migration, chemokine/cytokine secretion, T-cell activation, immune regulation, cytotoxicity (TRAIL/granzyme B), Treg induction, costimulatory/inhibitory molecule expression	[79,81,82,83,84,87]
Subpopulations	mCAFs, iCAFs, myoCAFs, ucCAFs	[36]	M0, M1, M2, F4/80^high, F4/80^low	[43,44]	B:Naive, CXCR4+, Follicular, Germinal center, Plasma; T: Th1, Th2, Th17, (n/i/o/x)Tregs, cytotoxic CD4+/CD8+	[51,54,59,60,65,70,72]	cDC1, cDC2, pDC, LCs, mregDC, DC3	[79,82,83,84,85]

## Abbreviations

The following abbreviations are used in this manuscript:

ACTA2	Actin alpha cardiac muscle 2
ADAM9	ADAM metallopeptidase domain 9
ARID5A	AT-rich interactive domain 5A
αSMA	Alpha smooth muscle actin
Bcl-XL	B-cell lymphoma-extra large
BIRC3	Baculoviral IAP repeat containing 3
BDCA1	Blood dendritic cell antigen 1
BDCA3	Blood dendritic cell antigen 3
BTLA	B- and T-lymphocyte attenuator
CAFs	Cancer-associated fibroblasts
cDC	Conventional dendritic cell
cDC1	Conventional dendritic cell type 1
cDC2	Conventional dendritic cell type 2
CD103	Cluster of differentiation 103
CD107a	Cluster of differentiation 107a
CD11c	Cluster of differentiation 11c
CD141	Cluster of differentiation 141
CD19	Cluster of differentiation 19
CD25	Cluster of differentiation 25
CD5	Cluster of differentiation 5
CD80	Cluster of differentiation 80
CD90	Cluster of differentiation 90
CD206	Cluster of differentiation 206
CCR1	C-C motif chemokine receptor 1
CCR2	C-C motif chemokine receptor 2
CCR7	C-C motif chemokine receptor 7
CXCL16	C-X-C motif chemokine ligand 16
CXCL9	C-X-C motif chemokine ligand 9
CXCL12	C-X-C motif chemokine ligand 12
CXCR3	C-X-C motif chemokine receptor 3
CXCR5	C-X-C motif chemokine receptor 5
DNGR-1	Dendritic cell natural killer receptor-1
EOMES	Eomesodermin
FCεR1	High-affinity immunoglobulin epsilon receptor 1
FLT3L	Fms-like tyrosine kinase 3 ligand
FAP	Fibroblast-activation protein
FOXP3	Forkhead box P3
GNLY	Granulysin
GM-CSF	Granulocyte-macrophage colony-stimulating factor
ICOS-L	Inducible co-stimulator ligand
ILT7	Immunoglobulin-like transcript 7
IFN-γ	Interferon gamma
IFNγ	Interferon gamma
IL-1β	Interleukin-1 beta
IL-6	Interleukin-6
IL-8	Interleukin-8
IL-10	Interleukin-10
IL-15	Interleukin-15
IL-12	Interleukin-12
IL-17	Interleukin-17
ID2	Inhibitor of DNA-binding 2
IDO	Indoleamine 2,3-dioxygenase
IDO1	Indoleamine 2,3-dioxygenase 1
IRF1	Interferon regulatory factor 1
IRF4	Interferon regulatory factor 4
IRF8	Interferon regulatory factor 8
IRF9	Interferon regulatory factor 9
KCNJ8	Potassium channel, inwardly rectifying subfamily J member 8
LAMP-1	Lysosomal-associated membrane protein 1
LAMP3	Lysosomal-associated membrane protein 3
LIPSTIC	Liposome-based short interfering RNA delivery technology for immunocellular targeting
MMP1	Matrix metalloproteinase 1
MMP3	Matrix metalloproteinase 3
MMPs	Matrix metalloproteinases
MRC1	Mannose receptor C-type 1
MARCKSL1	Myristoylated alanine-rich C-kinase substrate-like 1
MZB1	Marginal zone B-cell marker 1
NKG7	Natural killer cell granule protein 7
NK	Natural killer cells
OX40	Ox40 receptor
PD-1	Programmed cell death protein 1
PD-L1	Programmed cell death ligand 1
PD-L2	Programmed cell death ligand 2
PRDM1	PR domain containing 1
RELB	RelB proto-oncogene
RGS5	Regulator of G-protein signaling 5
RUNX3	Runt-related transcription factor 3
SDF-1	Stromal-derived factor 1
STAT1	Signal transducer and activator of transcription 1
STAT3	Signal transducer and activator of transcription 3
T-bet	T-box transcription factor
TCR	T-cell receptor
TILs	Tumor-infiltrating lymphocytes
TLR4	Toll-like receptor 4
TLR7	Toll-like receptor 7
TLR9	Toll-like receptor 9
TLS	Tumor lymphoid structures
TNF	Tumor necrosis factor
TNF-α	Tumor necrosis factor alpha
TME	Tumor microenvironment
TWIST1	Twist family bHLH transcription factor 1
TWIST2	Twist family bHLH transcription factor 2
XCL1	C-X-C motif chemokine ligand 1
XCL2	C-X-C motif chemokine ligand 2
XCR1	C-X-C motif chemokine receptor 1
ZEB2	Zinc finger E-box binding homeobox 2
